# p21/Zbtb18 repress the expression of cKit to regulate the self-renewal of hematopoietic stem cells

**DOI:** 10.1093/procel/pwae022

**Published:** 2024-05-09

**Authors:** Nini Wang, Shangda Yang, Yu Li, Fanglin Gou, Yanling Lv, Xiangnan Zhao, Yifei Wang, Chang Xu, Bin Zhou, Fang Dong, Zhenyu Ju, Tao Cheng, Hui Cheng

**Affiliations:** State Key Laboratory of Experimental Hematology, National Clinical Research Center for Blood Diseases, Haihe Laboratory of Cell Ecosystem, Institute of Hematology & Blood Diseases Hospital, Chinese Academy of Medical Sciences & Peking Union Medical College, Tianjin 300020, China; CAMS Center for Stem Cell Medicine, PUMC Department of Stem Cell and Regenerative Medicine, Tianjin 300020, China; State Key Laboratory of Experimental Hematology, National Clinical Research Center for Blood Diseases, Haihe Laboratory of Cell Ecosystem, Institute of Hematology & Blood Diseases Hospital, Chinese Academy of Medical Sciences & Peking Union Medical College, Tianjin 300020, China; CAMS Center for Stem Cell Medicine, PUMC Department of Stem Cell and Regenerative Medicine, Tianjin 300020, China; State Key Laboratory of Experimental Hematology, National Clinical Research Center for Blood Diseases, Haihe Laboratory of Cell Ecosystem, Institute of Hematology & Blood Diseases Hospital, Chinese Academy of Medical Sciences & Peking Union Medical College, Tianjin 300020, China; CAMS Center for Stem Cell Medicine, PUMC Department of Stem Cell and Regenerative Medicine, Tianjin 300020, China; The Province and Ministry Co-sponsored Collaborative Innovation Center for Medical Epigenetics, Key Laboratory of Immune Microenvironment and Disease (Ministry of Education), Department of Cell Biology, Tianjin Medical University, Tianjin 300270, China; State Key Laboratory of Experimental Hematology, National Clinical Research Center for Blood Diseases, Haihe Laboratory of Cell Ecosystem, Institute of Hematology & Blood Diseases Hospital, Chinese Academy of Medical Sciences & Peking Union Medical College, Tianjin 300020, China; CAMS Center for Stem Cell Medicine, PUMC Department of Stem Cell and Regenerative Medicine, Tianjin 300020, China; State Key Laboratory of Experimental Hematology, National Clinical Research Center for Blood Diseases, Haihe Laboratory of Cell Ecosystem, Institute of Hematology & Blood Diseases Hospital, Chinese Academy of Medical Sciences & Peking Union Medical College, Tianjin 300020, China; CAMS Center for Stem Cell Medicine, PUMC Department of Stem Cell and Regenerative Medicine, Tianjin 300020, China; State Key Laboratory of Experimental Hematology, National Clinical Research Center for Blood Diseases, Haihe Laboratory of Cell Ecosystem, Institute of Hematology & Blood Diseases Hospital, Chinese Academy of Medical Sciences & Peking Union Medical College, Tianjin 300020, China; CAMS Center for Stem Cell Medicine, PUMC Department of Stem Cell and Regenerative Medicine, Tianjin 300020, China; State Key Laboratory of Experimental Hematology, National Clinical Research Center for Blood Diseases, Haihe Laboratory of Cell Ecosystem, Institute of Hematology & Blood Diseases Hospital, Chinese Academy of Medical Sciences & Peking Union Medical College, Tianjin 300020, China; CAMS Center for Stem Cell Medicine, PUMC Department of Stem Cell and Regenerative Medicine, Tianjin 300020, China; State Key Laboratory of Experimental Hematology, National Clinical Research Center for Blood Diseases, Haihe Laboratory of Cell Ecosystem, Institute of Hematology & Blood Diseases Hospital, Chinese Academy of Medical Sciences & Peking Union Medical College, Tianjin 300020, China; State Key Laboratory of Experimental Hematology, National Clinical Research Center for Blood Diseases, Haihe Laboratory of Cell Ecosystem, Institute of Hematology & Blood Diseases Hospital, Chinese Academy of Medical Sciences & Peking Union Medical College, Tianjin 300020, China; CAMS Center for Stem Cell Medicine, PUMC Department of Stem Cell and Regenerative Medicine, Tianjin 300020, China; Key Laboratory of Regenerative Medicine of Ministry of Education, Institute of Aging and Regenerative Medicine, College of Life Science and Technology, Jinan University, Guangzhou 510632, China; State Key Laboratory of Experimental Hematology, National Clinical Research Center for Blood Diseases, Haihe Laboratory of Cell Ecosystem, Institute of Hematology & Blood Diseases Hospital, Chinese Academy of Medical Sciences & Peking Union Medical College, Tianjin 300020, China; CAMS Center for Stem Cell Medicine, PUMC Department of Stem Cell and Regenerative Medicine, Tianjin 300020, China; State Key Laboratory of Experimental Hematology, National Clinical Research Center for Blood Diseases, Haihe Laboratory of Cell Ecosystem, Institute of Hematology & Blood Diseases Hospital, Chinese Academy of Medical Sciences & Peking Union Medical College, Tianjin 300020, China; CAMS Center for Stem Cell Medicine, PUMC Department of Stem Cell and Regenerative Medicine, Tianjin 300020, China

**Keywords:** hematopoietic stem cells, self-renewal, p21, Zbtb18, cKit

## Abstract

The maintenance of hematopoietic stem cells (HSCs) is a complex process involving numerous cell-extrinsic and -intrinsic regulators. The first member of the cyclin-dependent kinase family of inhibitors to be identified, p21, has been reported to perform a wide range of critical biological functions, including cell cycle regulation, transcription, differentiation, and so on. Given the previous inconsistent results regarding the functions of p21 in HSCs in a p21-knockout mouse model, we employed *p21*-tdTomato (tdT) mice to further elucidate its role in HSCs during homeostasis. The results showed that p21-tdT^+^ HSCs exhibited increased self-renewal capacity compared to p21-tdT^−^ HSCs. Zbtb18, a transcriptional repressor, was upregulated in p21-tdT^+^ HSCs, and its knockdown significantly impaired the reconstitution capability of HSCs. Furthermore, p21 interacted with ZBTB18 to co-repress the expression of *cKit* in HSCs and thus regulated the self-renewal of HSCs. Our data provide novel insights into the physiological role and mechanisms of p21 in HSCs during homeostasis independent of its conventional role as a cell cycle inhibitor.

## Introduction

Hematopoietic stem cells (HSCs) are resident bone marrow (BM) stem cells responsible for maintaining the hematopoietic system throughout life (Cheng et al., 2020). A mounting number of studies show that the maintenance of HSCs is an intricately regulated process involving numerous cell-extrinsic and -intrinsic regulators (Itkin et al., 2016; Ito and Suda, 2014; Morrison and Scadden, 2014; Nakamura-Ishizu et al., 2014; Pietras et al., 2011; Pinho and Frenette, 2019; Sanjuan-Pla et al., 2013; Shin et al., 2014; Yamazaki et al., 2011; Ye et al., 2013). It is essential to understand the fundamental biology of HSCs to advance the development of HSC-based regenerative therapies.

p21, encoded by the *Cdkn1a* gene, is a polypeptide composed of 164 amino acids with a molecular weight of approximately 21 kDa (Dutto et al., 2015). It is the first member of the cyclin-dependent kinase (Cdk)-inhibitor (CKI) family to be recognized (Harper et al., 1993). Because of the lack of a defined three-dimensional structure, p21 is known as an intrinsically disordered protein (IDP). IDPs typically need to bind to specific functional proteins to participate in biological processes. And they often show "binding diversity" by adaptively folding their structures. Therefore, these highly dynamic IDPs fulfill myriad biological functions (Wang et al., 2011; Wright and Dyson, 2015). Due to this fundamental nature, p21 can interact with various targets, including transcription factors and kinases, to perform a wide range of critical biological functions (Dutto et al., 2015; Georgakilas et al., 2017; Karimian et al., 2016; Kreis et al., 2019; Mansilla et al., 2020). These functions encompass transcription, differentiation, cell cycle regulation, apoptosis, DNA repair, autophagy, the onset of senescence, and cell migration. For example, p21 has been reported to be associate with the E2F-1 transcription factor repressing the *Wnt4* gene in keratinocytes (Delavaine and La Thangue, 1999; Devgan et al., 2005). In addition to its multifaceted cellular functionality, p21 plays opposite roles depending on the cellular context and its subcellular localization (nucleus or cytoplasm) and expression levels (Dutto et al., 2015; Georgakilas et al., 2017; Karimian et al., 2016; Kreis et al., 2019; Mansilla et al., 2020). Even after about 30 years, p21 remains a challenging and fascinating protein, more complex than initially identified as a universal CKI.

Initially, the results from p21-knockout mice with a 129/sv background suggested p21 was a key molecule to restrict the cell cycle of HSCs (Cheng et al., 2000). Its absence led to increased cell cycling, resulting in stem-cell exhaustion. This initial study opened the door for studies on the roles of CKIs in HSCs and more generally in stem-cell biology (Cheng, 2004; Janzen et al., 2006; Qiu et al., 2004; Stier et al., 2003), the mice with C57/B6 background did not exhibit altered cell cycle status of HSCs and no significant differences were detected in the number or self-renewal of HSCs in p21-knockout mice in serial transplantations and 5-fluorouracil treatment in other studies (Matsumoto et al., 2011; van Os et al., 2007). Nevertheless, HSC self-renewal in mice lacking p21 was found to be impaired under stressful conditions, such as DNA damage induced by exposure to 5-fluorouracil and γ-irradiation (Cheng et al., 2000; van Os et al., 2007). These ambiguous results highlight the need for further studies to precisely investigate the function of p21 in HSCs, particularly under homeostatic conditions.

Furthermore, p21 global knockout mice, despite being widely used, could not uncover the real role of p21 with several defects in HSCs. First, p21's functions vary with its expression levels (LaBaer et al., 1997). For example, at low concentrations, p21 enhances the activity of the CDK4-cyclinD kinase complex; conversely, at higher concentrations, it inhibits this activity. Hence, the global deletion of p21 is inadequate for a complete understanding of its role in HSCs at physiological concentrations during homeostasis. Second, the function of HSCs is intricately regulated by a complex interplay of cell-intrinsic and cell-extrinsic factors. Considering that p21 is widely expressed, the global deletion of p21 may influence other cells in the hematopoietic microenvironment, subsequently impacting the functionality of HSCs. It is clearly unreasonable to attribute all the changes solely to the function of p21 in HSCs. To overcome these inherent limitations, *p21*-tdTomato (tdT) mice were used in this study, allowing us to distinguish between p21-tdT^−^ and p21-tdT^+^ HSCs via the fluorescence intensity of tdTomato, without p21 knockout (Li et al., 2018). Our results suggested that p21-tdT^+^ HSCs possessed increased self-renewal capacity. p21 was shown to interact with ZBTB18, a transcriptional repressor, to repress the expression of *cKit*, a mechanism that underlies the enhanced repopulating ability of p21-tdT^+^ HSCs. These findings provide novel insights into the physiological function of p21 in HSCs during normal, unperturbed homeostasis.

## Results

### p21-tdT^+^ HSCs were in an inactive state of cell division

**Figure 1.**
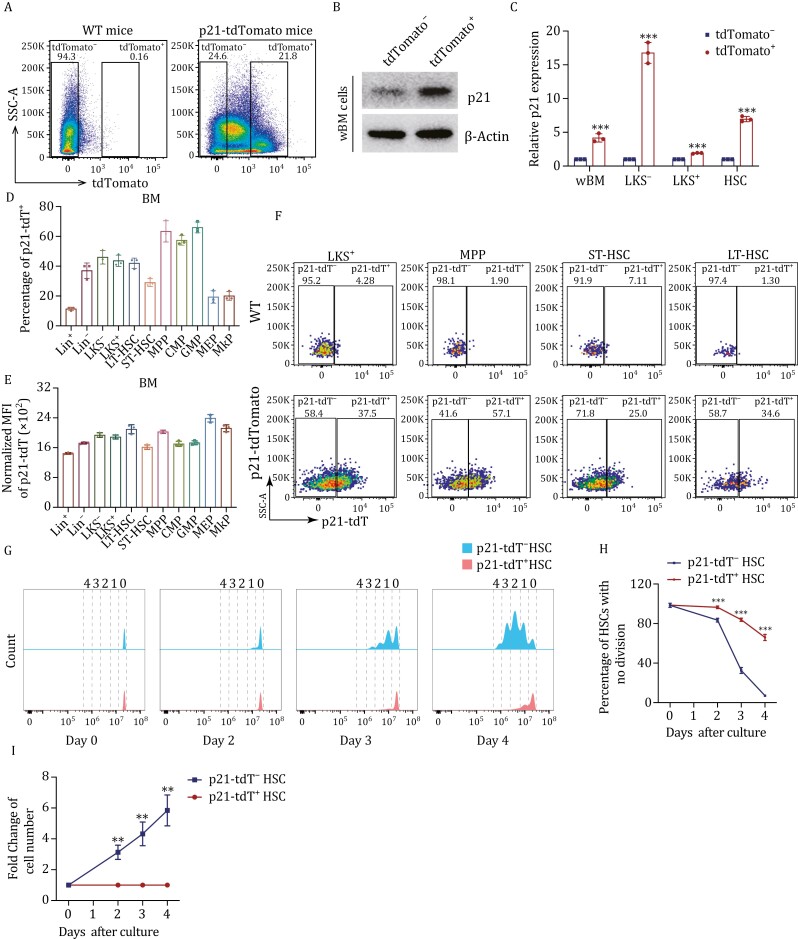
p21-tdT^+^ HSCs were in an inactive state of cell division. (A) Gating strategy used to isolate tdTomato^−^ and tdTomato^+^ whole bone marrow (wBM) cells from *p21*-tdTomato mice. (B) Western blot analysis of the expression of p21 in the tdTomato^−^ and tdTomato^+^ wBM cells from *p21*-tdTomato mice. (C) qRT-PCR analysis of *p21* expression in indicated BM cell populations (*n* = 3 samples per group, 25 mice used for cell isolation). (D and E) Quantification of percentages of p21-tdT^+^ cells (D) and mean fluorescence intensity (MFI) of p21 expression (E) in indicated BM cell populations (*n* = 3 per group). (F) Gating strategy of p21-tdT^+^ cell populations in HSPCs from wild type (WT) and *p21*-tdTomato mice (*n* = 3 per group). (G) p21-tdT^−^ and p21-tdT^+^ cells stained with CellTrace Violet dye were cultured for 4 days, and the cell division kinetics was measured using the fluorescence intensity of CellTrace Violet by flow cytometry (*n* = 3 per group, 30 mice used for cell isolation). (H) Percentages of indicated cell populations with no division derived from p21-tdT^−^ HSCs or p21-tdT^+^ HSCs within the 4-day period *in vitro* culture (*n* = 3 per group, 30 mice used for cell isolation). (I) Foldchange of the progeny cell number from p21-tdT^−^ HSCs or p21-tdT^+^ HSCs within the 4-day period *in vitro* culture (*n* = 3 per group, 30 mice used for cell isolation). Data are represented as mean ± SD. **, *P* < 0.01; ***, *P* < 0.001; unpaired two-tailed Student's *t* test for (C, H, and I).

Given the multifaceted functions of p21 at different expression levels (Dutto et al., 2015; Georgakilas et al., 2017; Karimian et al., 2016; Kreis et al., 2019; Mansilla et al., 2020), it does not make sense to use global p21-knockout mice to study the physiological role of p21 in HSCs during homeostasis. Therefore, we employed *p21*-tdTomato (tdT) mice to focus on p21-tdT^−^ and p21-tdT^+^ HSCs (Li et al., 2018) (Fig. S1A). First, we isolated tdTomato^−^ and tdTomato^+^ cells from total nucleated cells in the BM of *p21*-tdTomato mice using fluorescence-activated cell sorting (FACS) and assessed their levels of p21 protein (Fig. 1A). Western blot analysis confirmed that tdTomato^+^ cells exhibited a higher level of p21 protein compared to tdTomato^−^ cells (Fig. 1B). We then collected tdTomato^−^ and tdTomato^+^ cells from whole (w)BM, Lineage^–^cKit^+^Sca1^−^ (LKS^−^), lineage^–^cKit^+^Sca1^+^ (LKS^+^), and CD34^−^LKS^+^ cells (HSCs) and analyzed their expressions of p21. The results showed that p21 expression was higher in tdTomato^+^ cells than tdTomato^−^ cells (Fig. 1C). In all, these data indicated that the expression levels of p21 could be distinguished using the fluorescence intensity of tdTomato.

In addition, the p21 expression in p21-tdT^+^ LKS^+^ cells was about twice as high as that in p21-tdT^−^ LKS^+^ cells, while that in p21-tdT^+^ LKS^−^ cells was approximately eighteen times higher than that in p21-tdT^−^ LKS^−^ cells (Fig. 1C). Thus, the p21 expressions of different p21-tdT^+^ cell populations exhibited fold-change differences compared to p21-tdT^−^ cells (Fig. 1C). To further elucidate p21 expression in each population, we quantified the percentage of p21-tdT^+^ cells and the mean fluorescence intensity (MFI) of p21 expression in mature cells and hematopoietic stem and progenitor cells (HSPCs) from the BM (Figs. 1D–F and S1B–E). Our findings revealed that, on average, the proportions of p21-tdT^+^ cells were 0.7%, 11.5%, 6.8%, and 1.8% within CD3^+^, B220^+^, CD11b^+^, and Ter119^+^ cells, respectively (Fig. S1D and S1E). Furthermore, the overall proportion of p21-tdT^+^ cells among all mature cells (Lineage^+^, Lin^+^) is approximately 11.3%. Worth noting, the ratio of p21 expression was higher in multipotent progenitors (MPPs), common myeloid progenitors (CMPs), and granulocyte-macrophage (GMPs) than in Lin^+^, Lin^−^, LKS^−^, LKS^+^, long-term (LT)-HSCs, short-term (ST)-HSCs, megakaryocyte-erythrocyte progenitors (MEPs), and megakaryocyte progenitors (MkPs) (Fig. 1D). Overall, the p21-tdT^+^ percentage and p21-tdT MFI varied across the different cell populations. Moreover, we found the expression of p21 to be lower in LT-HSCs than in certain progenitor cells, such as GMPs (Fig. 1E). This result corrected the previous traditional perception that p21 is expressed at the highest levels in HSCs.

p21 is recognized as a classic cell cycle inhibitor, however, the previous research about its role in HSCs are inconsistent (Cheng et al., 2000; Foudi et al., 2009; Matsumoto et al., 2011; van Os et al., 2007). Therefore, to further gain the comprehensive understanding of the actual cell division kinetics in HSCs, we recorded the cell division number of p21-tdT^−^ and p21-tdT^+^ HSCs stained with CellTrace Violet *in vitro* culture for 4 days (Figs. 1G and S1F). Notably, p21-tdT^−^ HSCs divided dramatically and almost all of them had completed at least one division; however, about 60% of p21-tdT^+^ HSCs remained undivided on Day 4 (Fig. 1H). Furthermore, there was a significantly increased fold-change of the progeny cell number in p21-tdT^−^ HSCs compared to p21-tdT^+^ HSCs within the 4-day period (Fig. 1I). These results directly reflected that p21-tdT^+^ HSCs underwent fewer cell divisions than p21-tdT^−^ HSCs, suggesting that p21-tdT^+^ HSCs were in an inactive state of the cell cycle actually. These results elucidate the role of p21 in HSCs during homeostasis, resolving previous conflicting findings and establishing a robust foundation for the applicability of *p21*-td Tomato mice. Moreover, considering its multifaceted cellular functionality as an intrinsically disordered protein (IDP), our subsequent goal is to uncover new insights into the specific functions and mechanisms of p21 in HSCs.

### p21-tdT^+^ HSCs possess enhanced self-renewal capacity

**Figure 2.**
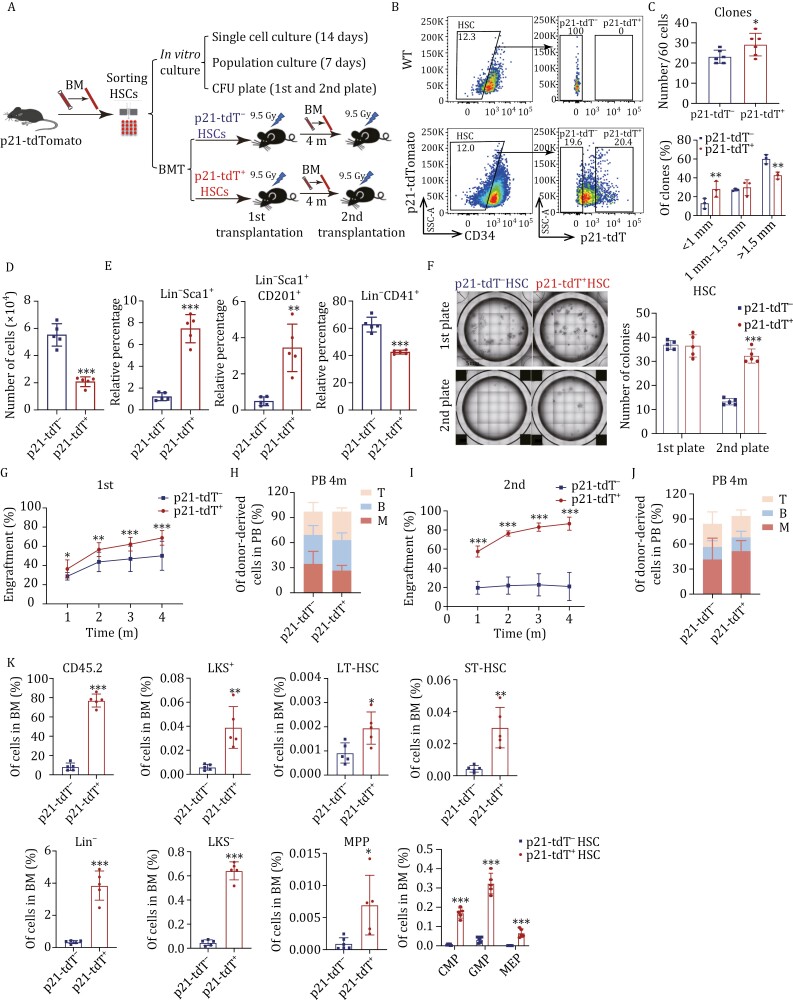
p21-tdT^+^ HSCs possess enhanced self-renewal capacity. (A) Schematic representation of *in vitro* culture and serial transplantation assays of p21-tdT^−^ or p21-tdT^+^ HSCs from *p21*-tdTomato mice (CD45.2) transplanted into B6.SJL mice (CD45.1). (B) Gating strategy used to sort p21-tdT^−^ or p21-tdT^+^ HSCs from *p21*-tdTomato mice. (C) Quantification of single-cell-formed clones and percentages of clones of different sizes from p21-tdT^−^ or p21-tdT^+^ HSCs (*n* = 6 per group). (D) 4500 p21-tdT^−^ and p21-tdT^+^ HSCs were double-FACS sorted into liquid culture medium. After 7 days in culture, the total cell output was quantified (*n* = 5 per group, 20 mice used for cell isolation). (E) Percentages of indicated cell populations derived from p21-tdT^−^ HSCs or p21-tdT^+^ HSCs after 7 days in liquid culture (*n* = 5 per group, 20 mice used for cell isolation). (F) Representative images and histograms of colonies from p21-tdT^−^   and p21-tdT^+^ HSCs are shown. Scale bars, 5 mm. 300 p21-tdT^−^ and p21-tdT^+^ HSCs were double-FACS sorted into methylcellulose medium, and total colony output was scored on Day 8. After 8 days of primary culture, 3,000 cells from each culture were replated into fresh methylcellulose medium, and total colonies were again scored on Day 8 (*n* = 5 per group). (G) Percentages of total donor CD45.2 cells in mouse peripheral blood (PB) over time after primary competitive transplantation (*n* = 5 mice pooled for donor cell isolation; *n* = 10 recipient mice per condition). (H) Percentages of lineage reconstitution in the PB in the 4th month post-transplantation (*n* = 10 recipient mice per condition). (I) Percentages of total donor CD45.2 cells in PB over time after secondary transplantation (*n* = 5 mice pooled for donor cells; *n* = 5 recipient mice per condition). (J) Percentages of lineage reconstitution in the PB in the 4th month post-transplantation (*n* = 5 recipient mice per condition). (K) Percentages of donor CD45.2 cells and donor-derived indicated cells in the BM of recipient mice in 4th month after secondary transplantation (*n* = 5 recipient mice per condition). Data are represented as mean ± SD. *, *P* < 0.05; **, *P* < 0.01; ***, *P* < 0.001; unpaired two-tailed Student's *t* test for (C–G, I, and K).

To further elucidate the function of p21 in HSCs, we performed *in vitro* culture and serial transplantation experiments after sorting p21-tdT^−^ and p21-tdT^+^ HSCs from the BM of *p21*-tdTomato mice (Fig. 2A and 2B). Following 14 days of single-cell culture, we found a slight difference in the total number of clones between p21-tdT^−^ and p21-tdT^+^ HSCs (Fig. 2C). However, there was a notable difference in clone size and type: p21-tdT^+^ HSCs produced smaller clones composed of homogeneous cells, whereas p21-tdT^−^ HSCs generated larger clones consisting of cells of various sizes (Figs. 2C, S2A and S2B). To further assess their proliferative capacity, 4,500 p21-tdT^−^ and p21-tdT^+^ HSCs were seeded into a serum-free expansion medium, and the hematopoietic cell output and populations were evaluated after 7 days. p21-tdT^+^ HSCs yielded 2.7-fold fewer cells than p21-tdT^−^ HSCs (Figs. 2D and S2C). Moreover, p21-tdT^+^ HSCs produced 6.2-fold more Lin^−^Sca1^+^ cells, 7.1-fold more Lin^−^Sca1^+^ CD201^+^ cells, and 1.5-fold fewer Lin^−^CD41^+^ cells compared with p21-tdT^−^ HSCs (Fig. 2E). In addition, to confirm their colony-forming ability, we cultured 300 p21-tdT^−^ and p21-tdT^+^ HSCs in methylcellulose media for 8–10 days. Although p21-tdT^−^ and p21-tdT^+^ HSCs generated similar numbers of colonies in the primary plates (Fig. 2F), p21-tdT^+^ HSCs generated higher frequencies of colony-forming unit granulocytes (CFU-G) and fewer colony-forming unit granulocytes/erythrocytes/macrophages/megakaryocytes (CFU-GEMM) (Fig. S2D). Furthermore, when 3,000 cells from the primary methylcellulose cultures were secondarily plated, p21-tdT^+^ HSCs produced more colonies compared to p21-tdT^−^ HSCs (Fig. 2F). Overall, these results showed that p21-tdT^+^ HSCs maintained their self-renewal and less differentiated status.

To assess the regenerative capacity of p21-tdT^−^ and p21-tdT^+^ HSCs, we transplanted 350 donor cells (CD45.2) accompanied by competitor cells (CD45.1) into irradiated recipients (CD45.1) (Fig. 2A and 2B). During 4 months posttransplant, the mice transplanted with p21-tdT^+^ HSCs displayed increased total donor cell peripheral blood (PB) chimerism compared with mice transplanted with p21-tdT^−^ HSCs (Fig. 2G). Given that homing is a crucial step for successful engraftment after HSC transplantation, we assessed the homing ability of both p21-tdT^−^ and p21-tdT^+^ HSC subsets. The results revealed no significant difference in homing ability between the two subsets (Fig. S2E and S2F). In addition, we also observed no significant difference in the LT-HSC output in the BM of recipients between the two groups at 4th-month postprimary transplantation (Fig. S2G and S2H). Moreover, primary transplantations of p21-tdT^−^ and p21-tdT^+^ HSCs yielded comparable lineage reconstitution in the PB of recipients (Fig. 2H). Furthermore, in the secondary transplantation assays using BM cells isolated from primary recipient mice 4 months post-transplantation, we observed the significantly increased engraftment of donor hematopoietic cells in the PB of recipients transplanted with p21-tdT^+^ HSCs compared to those transplanted with p21-tdT^−^ HSCs (Fig. 2I). There was no significant difference in the lineage reconstitution in the PB between the two groups (Fig. 2J). Moreover, mice transplanted with p21-tdT^+^ HSCs displayed increased percentages of donor-derived LT-HSCs, ST-HSCs as well as progenitors in the BM compared with p21-tdT^−^ HSCs (Figs. 2K and S2H). In summary, the results support the notion that p21-tdT^+^ HSCs possess enhanced long-term engraftment potential and increased self-renewal capacity compared to p21-tdT^−^ HSCs.

### p21-tdT^−^ and p21-tdT^+^ HSCs can convert to each other

**Figure 3.**
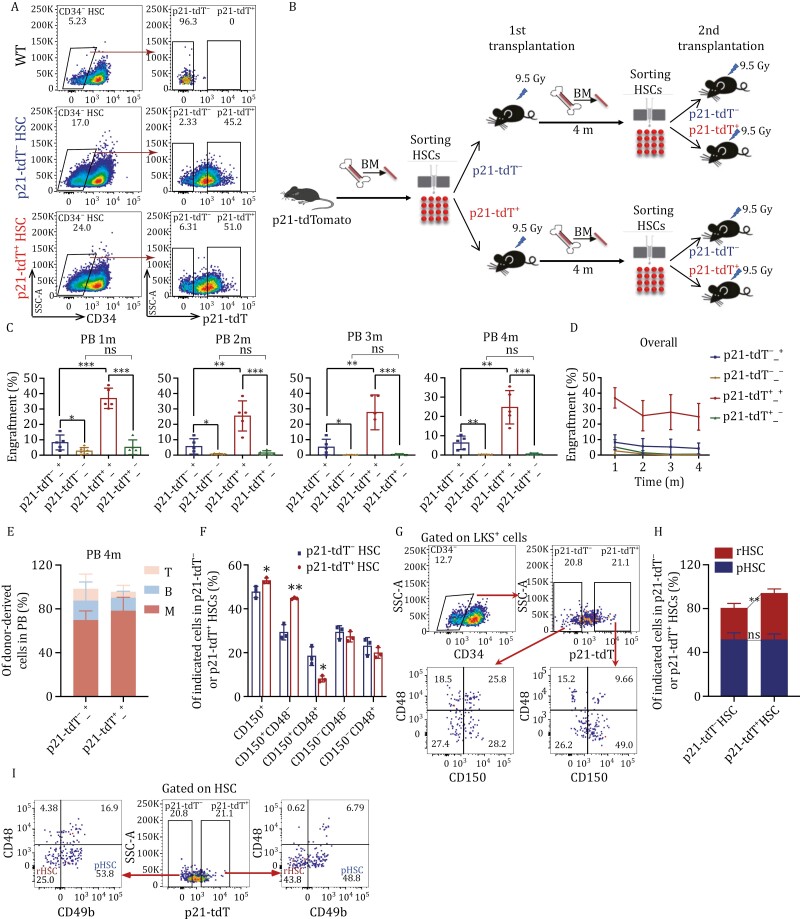
p21-tdT^−^ and p21-tdT^+^ HSCs can convert to each other. (A) Gating strategy for secondary transplantation of p21-tdT^−^ or p21-tdT^+^ HSCs from primary recipients transplanted with p21-tdT^−^ or p21-tdT^+^ HSCs (*n* = 7 mice pooled for donor cell isolation; *n* = 5 recipient mice per condition). (B) Schematic representation of primary and secondary competitive transplantation assays of p21-tdT^−^ or p21-tdT^+^ HSCs from *p21*-tdTomato mice and primary recipients. (C–E) Percentages of total donor CD45.2 cells in the PB in each month after secondary transplantation (C and D), and percentages of lineage reconstitution in the PB in the 4th month post-transplantation (E, *n* = 5 per group). (F and G) Percentages of surface marker expressions of CD150 and CD48 (F) and representative flow cytometric analysis (G) on p21-tdT^−^ and p21-tdT^+^ HSCs from *p21*-tdTomato mice (*n* = 3 per group). (H and I) The histograms (H) showing the percentages of rHSCs/pHSCs and representative flow cytometric analysis (I) in p21-tdT^−^ and p21-tdT^+^ HSCs (*n* = 3 per group). Data are represented as mean ± SD. ns, no significance; *, *P* < 0.05; **, *P* < 0.01; ***, *P* < 0.001; unpaired two-tailed Student's *t* test for (C, F and H).

Considering the increased self-renewal capacity of p21-tdT^+^ HSCs, we further investigated whether p21-tdT^+^ HSCs reside above p21-tdT^−^ HSCs in the hematopoietic hierarchical model. Flow cytometric analysis revealed that p21-tdT^+^ HSCs regenerated both p21-tdT^−^ and p21-tdT^+^ HSCs in transplanted recipient mice. Similarly, recipients of p21-tdT^−^ HSCs also exhibited the presence of both p21-tdT^−^ and p21-tdT^+^ HSCs in their BM in 4th month posttransplant (Fig. 3A). These findings suggest bidirectional conversion of the cells, with p21-tdT^+^ HSCs generating p21-tdT^−^ HSCs and *vice versa*. Given that both p21-tdT^−^ and p21-tdT^+^ HSCs can generate p21-tdT^+^ HSCs, the question as to which source of p21-tdT^+^ HSCs possess enhanced engraftment capacity arises. Additionally, further investigations were required to determine whether p21-tdT^+^ HSCs retain their increased self-renewal capacity compared to p21-tdT^−^ HSCs after experiencing the transplantation stress.

To detect the reconstitution capacity of p21-tdT^−^ and p21-tdT^+^ HSCs derived from primary recipients transplanted with the original p21-tdT^−^ or p21-tdT^+^ HSCs, we sorted the four subsets of HSCs in 4th-month post-transplant and transplanted secondary recipients with 350 donor cells (CD45.2) accompanied with competitor cells (CD45.1) into irradiated recipients (CD45.1) (Fig. 3B). Remarkably, p21-tdT^+^ HSCs showed increased reconstitution in secondary recipients compared with p21-tdT^−^ HSCs from the same primary recipients that were transplanted with either p21-tdT^−^ HSCs or p21-tdT^+^ HSCs (Fig. 3C). This finding is consistent with the freshly sorted p21-tdT^−^ and p21-tdT^+^ HSCs from *p21*-tdTomato mice (Fig. 2G and 2I). These data suggest that p21-tdT^+^ HSCs have higher self-renewal potential compared with p21-tdT^−^ HSCs under homeostatic conditions and transplantation stress. In addition, no significant difference was observed between the two groups of p21-tdT^−^ HSCs generated from the original p21-tdT^−^ or p21-tdT^+^ HSCs, yet both subsets exhibited comparably poor engraftment of secondary recipients (Fig. 3C and 3D).

Of note, p21-tdT^+^ HSCs derived from the original p21-tdT^+^ HSCs of primary recipients had a robust long-term repopulating capacity compared with p21-tdT^+^ HSCs from the original p21-tdT^−^ HSCs (Fig. 3C and 3D). There was no significant difference in lineage reconstitution in the PB of secondary recipients across the two subsets, suggesting that the effect of p21 was not directly linked to lineage-specific differentiation biases (Fig. 3E). The significant difference in the reconstitution capacity of secondary transplantation was directly attributed to the origin of the HSCs: p21-tdT^−^ or p21-tdT^+^.

Given that the surface expression of CD150 and the absence of CD48 expression can enrich HSCs (Kiel et al., 2005; Morita et al., 2010), we used another HSC-marking strategy in which we found increased CD150^+^ and CD150^+^CD48^−^ cell populations and decreased CD150^+^CD48^+^ cell population in p21-tdT^+^ HSCs compared with p21-tdT^−^ HSCs in the BM of *p21*-tdTomato mice (Fig. 3F and 3G). Additionally, previous studies have reported that a subset of reserve HSCs (rHSCs) display a greater capacity to replenish the HSC pool following myeloablation compared to primed HSCs (pHSCs) (Zhao et al., 2019). Then we labeled p21-tdT^−^ and p21-tdT^+^ HSCs with CD49b and CD48 markers. We observed no significant difference in the percentages of pHSCs between the two groups. However, the proportion of rHSCs in p21-tdT^+^ HSCs was higher than that within p21-tdT^−^ HSCs (Fig. 3H–I). These results are consistent with our conclusion that p21-tdT^+^ HSCs exhibit enhanced long-term reconstitution and self-renewal capabilities.

### Zbtb18 is highly expressed in p21-tdT^+^ HSCs

**Figure 4.**
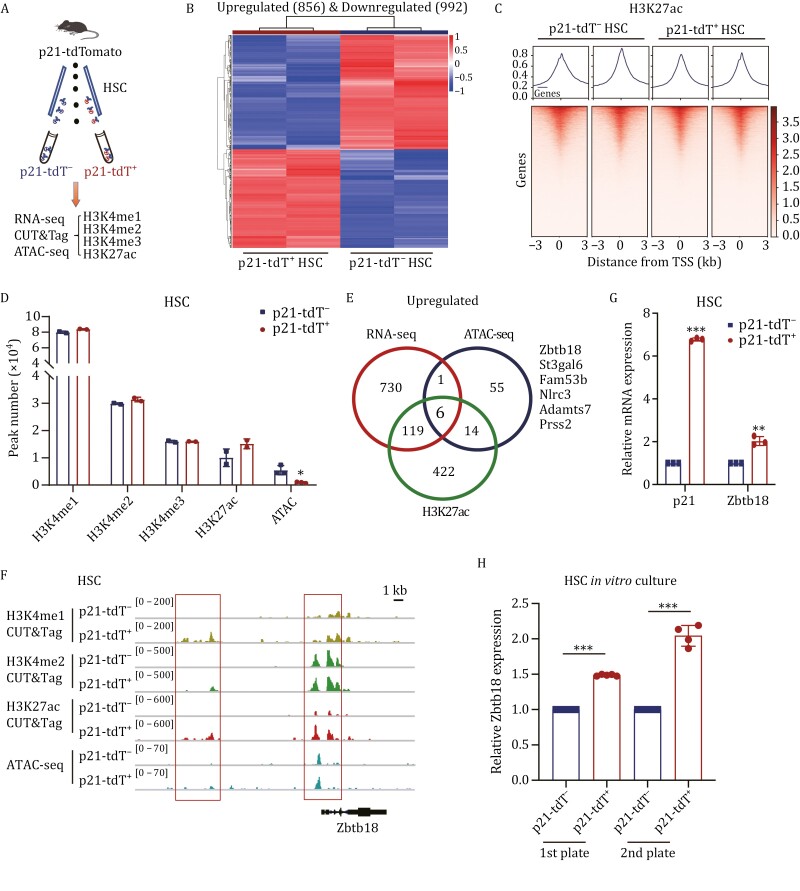
Zbtb18 is highly expressed in p21-tdT^+^ HSCs. (A) Experimental flow chart for sorting p21-tdT^−^ and p21-tdT^+^ HSCs from BM of *p21*-tdTomato mice to perform RNA-seq, ATAC-seq, and CUT&Tag (60 mice used for cell isolation). (B) Heatmap of differentially expressed genes (DEGs) in RNA-seq results for p21-tdT^−^ and p21-tdT^+^ HSCs from *p21*-tdTomato mice. (C) Transcription start site (TSS) profile and heatmap of CUT&Tag sequencing results showing binding of H3K27ac histone modification in p21-tdT^−^ and p21-tdT^+^ HSCs. (D) Quantification of the peak number of H3K4me1, H3K4me2, H3K4me3, and H3K27ac histone modifications in CUT&Tag-seq and ATAC-seq results in p21-tdT^−^ and p21-tdT^+^ HSCs from *p21*-tdTomato mice (*n* = 2 replicates). (E) Number of overlapping upregulated DEGs and peak genes in p21-tdT^+^ HSCs according to RNA-seq, ATAC-seq, and CUT&Tag-seq of H3K27ac. (F) IGV software visualization of CUT&Tag results for H3K4me1, H3K4me2, and H3K27ac and ATAC-seq in relation to the *Zbtb18* gene cis-regulate region in p21-tdT^−^ and p21-tdT^+^ HSCs from *p21*-tdTomato mice. (G) qRT-PCR analysis of *p21* and *Zbtb18* expression in p21-tdT^−^ and p21-tdT^+^ HSCs from *p21*-tdTomato mice (*n* = 3 per group). (H) qRT-PCR analysis of *Zbtb18* expression in p21-tdT^−^ and p21-tdT^+^ HSCs after primary plate and secondary plate (*n* = 4–6 per group). Data are represented as mean ± SD. *, *P* < 0.05; **, *P* < 0.01; ***, *P* < 0.001; unpaired two-tailed Student's *t* test for (D, G and H).

To uncover the molecular mechanisms underlying the increased repopulating capacity of p21-tdT^+^ HSCs, we performed RNA-sequencing (RNA-seq) analysis of p21-tdT^−^ and p21-tdT^+^ HSCs sorted from the BM of *p21*-tdTomato mice (Fig. 4A). Compared with p21-tdT^−^ HSCs, 856 genes were upregulated and 992 genes (Table S1) were downregulated in p21-tdT^+^ HSCs (Fig. 4B).

To further gain insight into the comprehensive transcriptional characterization of the two subsets, we performed assay for ATAC-seq and CUT&Tag using antibodies against the histone modifications (Lara-Astiaso et al., 2014) H3K4me1, H3K4me2, H3K4me3, and H3K27ac on p21-tdT^−^ and p21-tdT^+^ HSCs (Figs. 4A, 4C, S3A and S3B). The results of the CUT&Tag profiles revealed no significant global differences in the two cell subsets (Figs. 4C and S3B). However, the results of peak calling analysis showed a slight increase in the peak number of H3K27ac, while the ATAC-seq peak number was significantly decreased in p21-tdT^+^ HSCs (Fig. 4D). ATAC-seq peak calling analysis revealed 76 genes (Table S2) with increased accessibility and 4,403 genes with decreased accessibility in p21-tdT^+^ HSCs. As we all know, H3K27ac is well-recognized as a marker for active enhancers and a great indicator of transcriptional activation. However, decreased ATAC-seq peak number suggested the less accessible chromatin and more transcriptionally repressed regions in p21-tdT^+^ HSCs. Therefore, these results support the idea that there may be some genes that are transcriptionally upregulated along with the repression of chromatin accessibility in p21-tdT^+^ HSCs.

To further explore the genes that are highly expressed in p21-tdT^+^ HSCs and functionally associated with chromatin accessibility regulation, we performed integrative analysis of the upregulated genes from RNA-seq, ATAC-seq, and CUT&Tag-seq of H3K27ac (Table S3), and six overlapping genes were identified (Fig. 4E). According to previous reports, Zbtb18, the only transcriptional repressor among these six genes, participates in the repression of chromatin accessibility (Aoki et al., 1998; Wang et al., 2023). In addition, it has been identified as a crucial role in the formation of the cerebral cortex and cerebellum (Baubet et al., 2012; Hirai et al., 2012; Okado et al., 2009; Xiang et al., 2012) and the regulation of the self-renewal in neural stem cells (Hirai et al., 2012). Therefore, we focused our efforts on the transcriptional repressor Zbtb18.

After visualization with Integrative Genomics Viewer (IGV), the results showed that the peaks from H3K4me1, H3K4me2, H3K27ac CUT&Tag and ATAC-seq at the cis-regulate region (CRR) of the Zbtb18 genome were increased in p21-tdT^+^ HSCs (Fig. 4F). Next, qRT-PCR assays showed that fresh and cultured p21-tdT^+^ HSCs had nearly a 1.5–2-fold increase in *Zbtb18* expression (Fig. 4G and 4H). Collectively, these results suggest that Zbtb18 is highly expressed in p21-tdT^+^ HSCs.

### *Zbtb18* knockdown impairs the functions of HSCs

**Figure 5.**
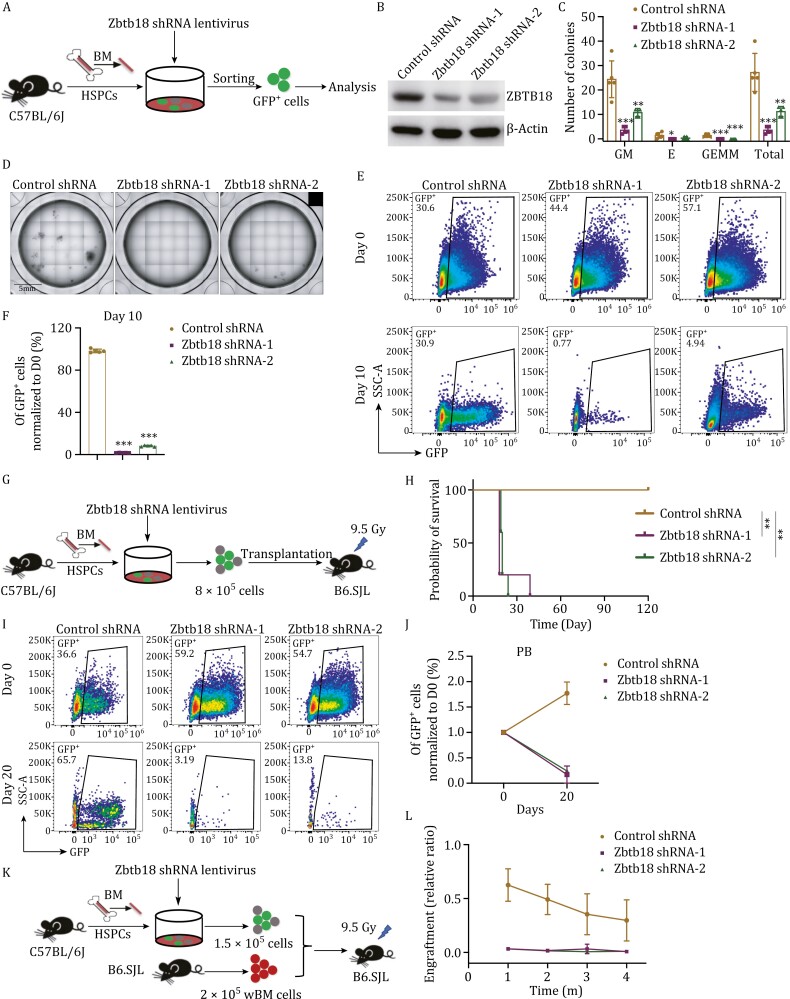
***Zbtb18*** knockdown impairs the functions of HSCs. (A) Schematic representation of the experimental procedures of gene knockdown. GFP-fused control or *Zbtb18* shRNA (*Zbtb18* shRNA-1, *Zbtb18* shRNA-2) lentiviruses were transduced into murine HSPCs, then GFP^+^ cells were sorted for subsequent assays. (B) Western blot analysis of ZBTB18 expression in murine GFP^+^ HSPCs transfected with control or *Zbtb18* shRNAs. (C and D) Colony counts from CFU assays of 500 GFP^+^ HSPCs well transfected with control or *Zbtb18* shRNAs and cultured for 7–10 days in complete methylcellulose-based medium (C, *n* = 5 per group). Representative images are shown in (D, *n* = 5 per group). Scale bars, 5 mm. (E and F) Representative flow cytometric analysis of the percentages of GFP^+^ HSPCs transfected with control or *Zbtb18* shRNAs cultured in serum-free expansion medium on Days 0 and 10 (E, *n* = 5 per group). The percentage of GFP^+^ HSPCs cultured on Day 10 normalized to Day 0 in groups of control and *Zbtb18* shRNAs (F, *n* = 5 per group). (G) Schematic representation of the transplantation assay of Zbtb18 knockdown. GFP-fused control or *Zbtb18* shRNA lentiviruses were transduced into donor C57BL/6J (CD45.2) murine HSPCs, which were injected into lethally irradiated (9.5 Gy) recipient B6.SJL (CD45.1) mice (*n* = 8 mice pooled for donor cell isolation; *n* = 5 recipient mice per group). (H) Survival curves of recipient mice transplanted with HSPCs transfected with control or *Zbtb18* shRNAs (*n* = 5 mice per group). (I and J) Representative flow cytometric analysis (I) and quantification of the percentages of donor GFP^+^ cells transfected with control or *Zbtb18* shRNAs in the PB of recipient mice on Day 20 posttransplant (J, *n* = 5 per group for transplantation, on Day 20 post-transplantation, the control group still had 5 mice, but *Zbtb18* shRNA-1 group had only two mice left, and *Zbtb18* shRNA-2 group had one remaining mouse). (K) Schematic representation of the competitive transplantation assay of Zbtb18 knockdown. GFP-fused control or *Zbtb18* shRNA lentiviruses were transduced into donor (CD45.2) murine HSPCs, which were injected into lethally irradiated (9.5 Gy) B6.SJL (CD45.1) recipient mice with 2 × 10^5^ (CD45.1) wBM cells (*n* = 6 mice pooled for donor cell isolation; *n* = 5 recipient mice per group). (L) Percentages of total donor CD45.2 GFP^+^ cells in the PB each month in recipient CD45.1 mice in the scrambled control, *Zbtb18* shRNA-1 and *Zbtb18* shRNA-2 groups (*n* = 5 recipient mice per group). Data are represented as mean ± SD. *, *P* < 0.05; **, *P* < 0.01; ***, *P* < 0.001; unpaired two-tailed Student's *t* test for (C and F); survival was analyzed by log-rank test for (H); two-way ANOVA with the Geisser–Greenhouse correction for (L).

To explore the functional impact of Zbtb18 on HSCs, we transduced GFP-expressing lentiviruses carrying *Zbtb18* short hairpin (sh)RNA into HSPCs from wild-type (WT) mice (C57BL/6J), and then performed *in vitro* CFU and *in vivo* transplantation assays (Fig. 5A). First, we confirmed that the two shRNA constructs exhibited high knockdown efficiencies at the protein level compared with the scrambled control (Fig. 5B). In CFU assays, *Zbtb18* knockdown significantly reduced the number of granulocyte-macrophage (CFU-GM) and CFU-GEMM colonies. The total number of colonies was also significantly decreased by 73% on average for the two groups of *Zbtb18*-knockdown cells compared with the control group (Fig. 5C and 5D). Additionally, we performed *in vitro* culture of GFP^+^ HSPCs transduced with *Zbtb18* shRNA and observed a significant decrease in the percentage of GFP^+^ cells on Day 10, suggesting that the cells with *Zbtb18* knockdown exhibited impaired proliferation (Fig. 5E and 5F).

Subsequently, we infected donor HSPCs with lentiviruses carrying *Zbtb18* shRNAs. After 48 h culture, 8 × 10^5^ cells were transplanted into lethally irradiated recipients without competitor cells (Fig. 5G). Seven out of 10 recipients from the two shRNA groups died within 20 days posttransplant (Fig. 5H), and the remaining three mice showed significantly decreased percentages of GFP^+^ cells in their PB compared with control recipients (Fig. 5I and 5J). Furthermore, we sorted GFP^+^ cells from the control, *Zbtb18*-shRNA-1, and *Zbtb18*-shRNA-2 groups and transplanted 1.5 × 10^5^ GFP^+^ cells into lethally irradiated recipients with 2 × 10^5^ competitor cells (Fig. 5K). Almost no reconstitution of GFP^+^ cells in the PB was observed over time among the recipients of the two shRNA groups (Fig. 5L).

Taken together, the results suggest that *Zbtb18* knockdown severely impairs the regeneration capability of HSCs, highlighting the critical role of Zbtb18 in maintaining HSC function. These findings are consistent with the higher expression of Zbtb18 and the enhanced engraftment potential of p21-tdT^+^ HSCs compared with p21-tdT^−^ HSCs.

### p21 interacts with ZBTB18 to repress the transcription of *cKit*

**Figure 6.**
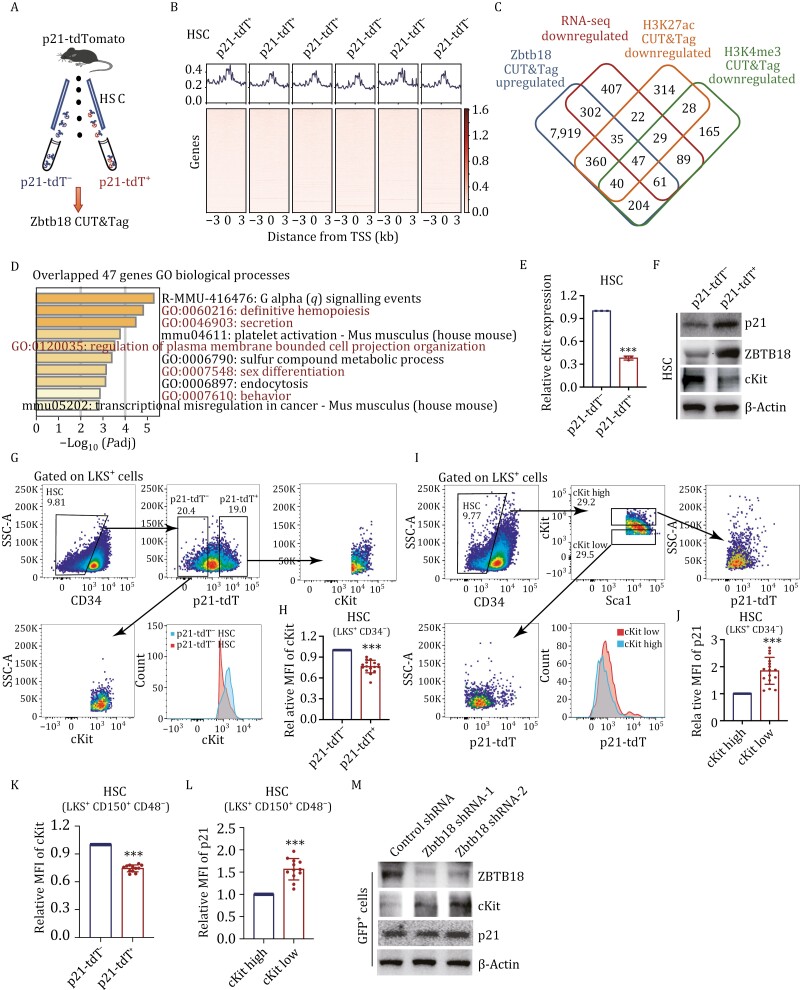
p21 interacts with ZBTB18 to repress the transcription of ***cKit***. (A) Experimental flow chart for sorting p21-tdT^−^ and p21-tdT^+^ HSCs from BM of p21-tdTomato mice to perform CUT&Tag assay against ZBTB18 antibodies (20 mice used for cell isolation). (B) TSS profile and heatmap of CUT&Tag sequencing results showing binding of ZBTB18. (C) Overlapping genes between increased peak genes in CUT&Tag sequencing analysis of Zbtb18 in p21-tdT^+^ HSCs from *p21*-tdTomato mice and downregulated genes in RNA-seq, and decreased peak genes in H3K4me3 and H3K27ac CUT&Tag sequencing analysis of p21-tdT^+^ HSCs. (D) GO term analysis of the 47 overlapping genes in (C), −log_10_ of the uncorrected *P* value on the x axis. (E) qRT-PCR analysis of *cKit* expression in p21-tdT^−^ and p21-tdT^+^ HSCs (*n* = 3 per group, 25 mice used for cell isolation). (F) Western blot analysis of expression of indicated proteins in p21-tdT^−^ and p21-tdT^+^ HSCs (30 mice used for cell isolation per experiment). (G and H) Representative gating strategy for flow cytometric analysis (G) and quantification of cKit mean fluorescence intensity (MFI) in p21-tdT^−^ and p21-tdT^+^ HSCs (LKS^+^CD34^−^) from p21-tdTomato mice (H, *n* = 16 per group). (I and J) Representative gating strategy of flow cytometric analysis (I) and quantification of p21 MFI in cKit-high and cKit-low HSCs (LKS^+^CD34^−^) (J, *n* = 16 per group). (K) Quantification of cKit MFI in p21-tdT^−^ and p21-tdT^+^ HSCs (LKS^+^CD150^+^CD48^−^) (*n* = 12 per group). (L) Quantification of p21 MFI in cKit-high and cKit-low HSCs (LKS^+^CD150^+^CD48^−^) (*n* = 12 per group). (M) Western blot analysis of expression of cKit, ZBTB18, and p21 in murine GFP^+^ HSPCs transfected with control or *Zbtb18* shRNAs. Data are represented as mean ± SD. ***, *P* < 0.001; unpaired two-tailed Student's *t* test for (E, H, J, K, and L).

To gain further insights into the molecular mechanisms underlying the Zbtb18-induced regulation of HSCs' repopulating capacity, we sorted p21-tdT^−^ and p21-tdT^+^ HSCs from *p21*-tdTomato mice and performed CUT&Tag assays using antibodies against ZBTB18 (Fig. 6A and 6B). Considering Zbtb18 is a transcriptional repressor, we conducted an integrated analysis of multiple sequencing data, including upregulated genes in Zbtb18 CUT&Tag and downregulated genes in RNA-seq and H3K4me3 and H3K27ac CUT&Tag to identify potential target genes in p21-tdT^+^ HSCs (Tables S3–5). A total of 47 genes overlapped (Table S6), including *cKit* (Fig. 6C). GO enrichment analysis showed that the top 10 biological processes of the 47 overlapping genes were predominantly cKit-related terms, such as definitive hemopoiesis (Fig. 6D).

According to previous studies, the cKit signaling pathway plays a critical role in the regulation of HSC functions, and dramatic phenotype changes are induced when the pathway is perturbed (Bosbach et al., 2012; Czechowicz et al., 2007; Deshpande et al., 2013; Ding et al., 2012; Krivtsov et al., 2006; McCulloch et al., 1964; Waskow et al., 2009). In addition, recent studies have demonstrated that HSCs with low expression levels of cKit exhibit enhanced self-renewal and long-term reconstitution potential compared to those with high levels of cKit (Grinenko et al., 2014; Matsuoka et al., 2011; Shah et al., 2023; Shin et al., 2014). Therefore, we detected the expression of cKit in p21-tdT^−^ and p21-tdT^+^ HSCs. The mRNA and protein levels of cKit in p21-tdT^+^ HSCs were decreased compared with p21-tdT^−^ HSCs (Fig. 6E and 6F). To further investigate the expression levels of cKit, we detected the MFI of cKit in p21-tdT^−^ and p21-tdT^+^ HSCs (LKS^+^CD34^−^) by flow cytometry. We also gated for cKit-low and cKit-high HSCs (LKS^+^CD34^−^) and detected the MFI of p21. The results demonstrated that p21-tdT^+^ HSCs expressed lower levels of cKit, while cKit-low HSCs expressed higher levels of p21 (Fig. 6G–J). The opposite expression pattern of p21 and cKit was also observed in LKS^+^CD150^+^CD48^−^ HSCs (Figs. 6K, 6L, S4A and S4B). These findings all suggest that p21-tdT^+^ HSCs with enhanced self-renewal ability express lower levels of cKit, which is consistent with the previous research. Moreover, *Zbtb18*-knockdown assays revealed the increased expression of cKit (Fig. 6M). Our results demonstrate that Zbtb18 represses the transcription of *cKit*.

**Figure 7.**
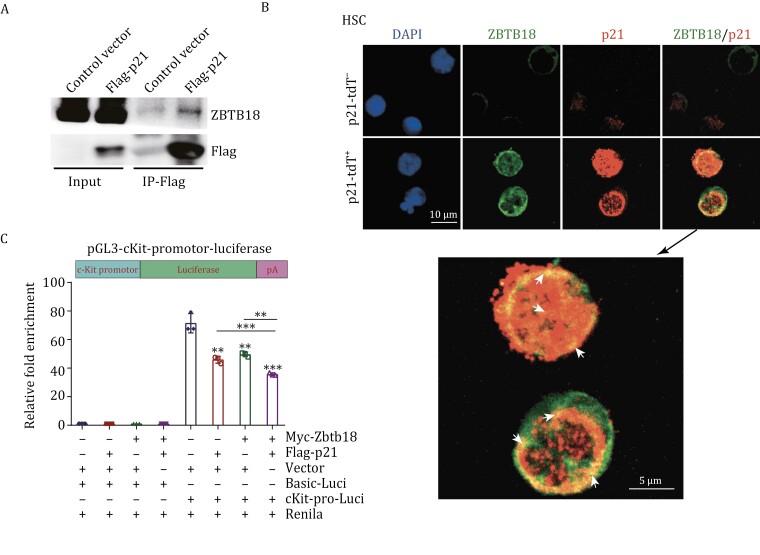
p21 interacts with ZBTB18 to repress the transcription of ***cKit***. (A) Western blot results showing enrichment of p21 in co-IP pulldown with ZBTB18 antibodies of 32D cells transfected with Flag-p21 plasmid. (B) Representative images of immunofluorescence labeling for ZBTB18 and p21 in p21-tdT^−^ and p21-tdT^+^ HSCs, scale bars, 10 μm (20 mice used for cell isolation per experiment). (C) The relative luciferase activity was determined by sequential normalization to the activity of Renilla and each corresponding control cells expressing pGL3-basic-luciferase activity are shown in (C, *n* = 3 per group). Schematic diagrams of pGL3-cKit-promotor-luciferase reporter constructs is exhibited. Data are represented as mean ± SD. **, *P* < 0.01; ***, *P* < 0.001; unpaired two-tailed Student's *t* test for (C).

To further explore the relationship between Zbtb18 and p21, we performed co-immunoprecipitation (co-IP) assays in 32D cells (Fig. 7A). The results exhibited that Zbtb18 could interact with p21. Moreover, immunofluorescence staining revealed more frequent co-localization of ZBTB18 and p21 in p21-tdT^+^ HSCs (Fig. 7B, arrows). These results showed an interaction between ZBTB18 and p21, suggesting a potential co-repressive function in target genes. To further test this, we established a pGL3-luciferase reporter containing the *cKit* promoter region and performed a reporter assay in 293T cells overexpressing p21, Zbtb18, or p21/Zbtb18 (Figs. 7C and S4C). The results showed that overexpressing p21 or Zbtb18 reduced the transcriptional activation of the *cKit* promoter-linked luciferase. Moreover, co-overexpression of p21 and Zbtb18 further decreased luciferase's activation compared to their individual overexpression. Altogether, these results suggest that p21 coordinates with ZBTB18 to repress the transcription of *cKit* and thus regulates the self-renewal and long-term regeneration capabilities of HSCs.

## Discussion

Faced with the past ambiguous results regarding the function of p21 in HSCs, we employed *p21*-tdTomato mice to distinguish p21-tdT^−^ and p21-tdT^+^ HSCs in homeostasis based on the fluorescence intensity of tdTomato, clarifying the role of p21 in HSCs. Further, our results showed that p21-tdT^+^ HSCs exhibited increased self-renewal capacity compared to p21-tdT^−^ HSCs, both in cell culture and transplantation assays. Subsequently, Zbtb18, a transcriptional repressor, was identified to be upregulated in p21-tdT^+^ HSCs through the integrative analysis of RNA, CUT&Tag, and ATAC sequencing data. Its knockdown significantly impaired the reconstitution capability and survival of HSCs, and Zbtb18 downregulated the expression of cKit which underlines the increased repopulating ability of p21-tdT^+^ HSCs. Furthermore, we revealed that p21 can interact with ZBTB18 to co-repress the transcription of *cKit* in HSCs, contributing to the regulation of HSC's self-renewal. Our data provide novel insights into the physiological function of p21 in HSCs different from its conventional role as a CKI during unperturbed homeostasis (Fig. S4D).

Due to the fundamental nature of IDP (Wang et al., 2011; Wright and Dyson, 2015), p21 can interact with various molecules (Dutto et al., 2015; Karimian et al., 2016; Kreis et al., 2019; Mansilla et al., 2020), such as E2F-1 (Delavaine and La Thangue, 1999; Devgan et al., 2005), to perform a wide range of functions, including transcription. However, previous studies on HSCs primarily focused on the interaction between p21 and the cyclin-CDK complexes, regulating the cell cycle to influence the self-renewal of HSCs (Cheng et al., 2000; Foudi et al., 2009; Matsumoto et al., 2011; van Os et al., 2007; Zou et al., 2011). Actually, the expression of p21 was not the highest in HSCs (Yamazaki et al., 2006; Zou et al., 2011), while p57 was highly expressed (Foudi et al., 2009; Matsumoto et al., 2011; Yamazaki et al., 2006; Yoshihara et al., 2007; Zou et al., 2011) and played a predominant role in the cell cycle regulation of HSCs among Cip/Kip family CDK inhibitors (p21, p27, p57) (Matsumoto et al., 2011; Zou et al., 2011). Deletion of p57 allele resulted in an increased expression of p27 and p18, but not p21 (Matsumoto et al., 2011; Zou et al., 2011). No significant difference in the cell cycle status was observed between LSK cells lacking p57 alone and those lacking both p21 and p57. However, additional ablation of p21 in a p57-null background resulted in a further decrease in the colony-forming activity of HSCs (Matsumoto et al., 2011). These results suggest that p21 may have functions in HSCs beyond the cell cycle regulation. Our results first provided the evidence that p21 can interact with a transcriptional repressor to regulate the self-renewal of HSCs, rather than binding to cyclin-CDK complexes. This study not only identified a novel binding protein for p21 but also discovered a new transcriptional regulatory role of p21 in HSCs independent of cell cycle regulation.

Accumulating evidences have shown that quiescence is not a uniform state, and the varying levels of quiescence are related to the number of cell divisions. It has been suggested that the bona fide HSCs divide only four to five times per lifetime during homeostasis and the self-renewal capacity of HSCs declines with each successive division (Bernitz et al., 2016; Foudi et al., 2009; Qiu et al., 2014; Wilson et al., 2008). These studies provide a comprehensive explanation of the secondary transplantation results of the four HSC subsets (Fig. 3B–E), combined with the data of the cell division kinetics (Figs. 1G–I and S1F). Particularly, p21-tdT^+^ HSCs derived from the original p21-tdT^+^ HSCs of primary recipients had a robust long-term repopulating capacity compared with p21-tdT^+^ HSCs from the original p21-tdT^−^ HSCs. This can be explained that p21-tdT^+^ HSCs derived from the original p21-tdT^+^ HSCs experienced fewer overall cell divisions, thereby retaining greater self-renewal ability. It has been widely accepted that the a relationship between the increased cell division number and the decreased self-renewal ability of HSCs. However, the mechanisms by which HSCs retain memory of their cell division history remain unclear. In our paper, as we distinguished HSCs based on the expression level of p21 and ATAC-seq data showed significant difference between p21-tdT^−^ and p21-tdT^+^ HSCs, we propose the hypothesis that p21 may play a role in the process of retaining memory about the cell division history of HSCs. Hence, we anticipate the enhanced reporter mice that can not only monitor the levels of p21 expression but also record the timing, duration, and frequency of p21 in the future. This advancement will facilitate a more profound comprehension of p21's role in the memory of cell division in HSCs.

In conclusion, we employed the fluorescence intensity of *p21*-tdTomato mice to distinguish between p21-tdT^−^ and p21-tdT^+^ HSCs in homeostasis and uncovered a novel role of p21 in regulating the physiological state of HSCs.

## Materials and methods

### Mice

C57BL/6J and B6.SJL mice were purchased from the animal facility of the State Key Laboratory of Experimental Hematology (SKLEH, Tianjin, China). *p21*-tdTomato mice with a C57BL/6J background were gifts from Dr. Bin Zhou. And the *p21*-tdTomato knock-in allele is generated by targeting tdTomato cDNA into the stop codon of p21 with the addition of 2A self-cleaving peptide sequence. All animal procedures were performed in compliance with the animal care guidelines and approved by the Institutional Animal Care and Use Committees of the SKLEH and the Institute of Hematology.

### Cell lines

293T and 32D cell lines were obtained from SKLEH's experimental pathology cell bank. All the cells were authenticated via examinations of their morphology and growth characteristics and were confirmed to be mycoplasma-free.

### Antibodies and reagents

The following antibodies were used in this study: FLAG (F1804; 1:2,000 for WB) and β-actin (A1978; 1:10,000 for WB) from Sigma–Aldrich; H3K4me3 (ab176877; 1:200 for CUT&Tag), H3K4me2 (ab176878; 1:200 for CUT&Tag), H3K4me3 (ab8580; 1:200 for CUT&Tag), and H3K27ac (ab6002; 1:200 for CUT&Tag) from Abcam; p21 (sc-6246; 1:1000 for WB) from Santa Cruz Biotechnology; and ZBTB18 (12714-1-AP; 1:1000 for WB and 1:100 for CUT&Tag) and cKit (18696-AP; 1:1000 for WB) from Proteintech. Recombinant murine SCF (250-03), recombinant murine TPO (315-14), recombinant murine IL-3 (213-13), and recombinant human EPO (100-64) were obtained from PeproTech.

### Plasmids and virus production

The LV-shRNA-GFP of *Zbtb18* lentiviruses was produced by GeneChem (Shanghai, China). For lentiviral production, the target plasmid together with pSPAX2 and pMD2G, was transfected into the 293T cell line using Lipofectamine 2000. The supernatant was harvested after 48 h and 72 h of culture and concentrated using an Amicon filter (100K NMWL; Millipore). The Flag-tagged *p21* was expressed using a pLVX-FLAG-IRES-Puro vector. The Myc-tagged *Zbtb18* was expressed using a pCMV6-Entry vector. The *cKit* promoter and luciferase were ligated into the pGL3-luciferase vector.

### Cell culture

For single-cell culture, HSCs collected from *p21*-tdTomato mice were directly sorted into 96-well plates containing α-MEM medium plus 10% FBS, mouse SCF (10 ng/mL), mouse TPO (10 ng/mL), mouse IL-3 (10 ng/mL), and human EPO (1 U/mL). After 14 days of culture, clones were counted and photographed. HSPCs were obtained from C57BL/6J mice and cultured in serum-free expansion medium (09650; Stem Cell) with mouse SCF (100 ng/mL) and mouse TPO (100 ng/mL). The 293T cells were maintained in DMEM (SH30243.01; Hyclone) supplemented with 10% FBS. The 32D cells were cultured in RPMI 1640 (A10491-01; Gibco) with 10% FBS and mouse IL-3 (10 ng/mL).

### Western blot

Western blot was performed as described previously (Yang et al., 2018). In brief, cellular extracts were harvested from cells and resuspended in 5× SDS–PAGE loading buffer. The boiled protein samples were then subjected to SDS-PAGE followed by immunoblotting with the appropriate primary antibodies and secondary antibodies.

### Flow cytometry

For hematopoietic stem and progenitor cells analysis, nucleated BM cells were stained with a lineage cocktail and antibodies against Sca1 (eBioscience; 25-5981-82; 1:200), cKit (eBioscience; 17-1171-82; 1:200), CD34 (eBioscience; 13-0341-82; 1:100), CD16/CD32 (eBioscience; 45-0161-82; 1:200), and CD135 (BioLegend; 135040; 1:400). The lineage cocktail included Gr1 (BioLegend; 108424; 1:400), Mac1 (BioLegend; 101226; 1:400), B220 (BioLegend; 103224; 1:400), CD4 (BioLegend; 100414; 1:400), CD8 (BioLegend; 100714; 1:400), CD3 (BioLegend; 100330; 1:400), and Ter119 (BioLegend; 116223; 1:400). In addition, nucleated BM cells were stained with the lineage cocktail and antibodies against Sca1, cKit, CD34, CD150 (BioLegend; 115904; 1:400), CD48 (BioLegend; 103432; 1:400) and CD49b (BioLegend; 103517; 1:400). For MPP analyses, nucleated BM cells were stained with the lineage cocktail, Sca1, cKit, CD150, and CD41 (eBioscience; 46-0411-82; 1:400). To sort p21-tdT^−^ and p21-tdT^+^ HSCs, HSPCs were enriched for flow cytometry using cKit magnetic beads (Miltenyi Biotec). The cells were subsequently stained with the lineage cocktail and cKit, Sca1, and CD34 antibodies. DAPI (D9542; 1 mg/mL; Sigma-Aldrich) was used to exclude dead cells. A modified LSR II flow cytometer with four lasers (355, 488, 561, and 633 nm) was used for the analysis, and an Aria III flow cytometer with four lasers (375, 488, 561, and 633 nm) was used for sorting. The analyses were performed using FACS Diva and FlowJo (TreeStar) software.

Immunophenotypes: HSC, Lin^−^cKit^+^Sca1^+^CD34^−^ or Lin^−^cKit^+^Sca-1^+^CD48^−^CD150^+^; LT-HSC, Lin^−^cKit^+^Sca1^+^Flk2^−^CD34^−^; ST-HSC, Lin^−^cKit^+^Sca1^+^Flk2^−^CD34^+^; MPP, Lin^−^cKit^+^Sca1^+^Flk2^+^CD34^+^; MkP, Lin^−^cKit^+^Sca1^−^CD41^+^CD150^+^; CMP, Lin^−^cKit^+^Sca1^−^Flk2^+^CD34^+^CD16/CD32^−^; GMP, Lin^−^cKit^+^Sca1^−^CD34^+^CD16/CD32^+^; MEP, Lin^−^cKit^+^Sca1^−^CD34^−^CD16/CD32^−^.

### Transplantation assays

The indicated cells were transplanted into lethally irradiated (9.5 Gy) mice via tail vein injection. PB was analyzed every one month for donor chimerism using flow cytometry. For competitive BM transplantation, 350 p21-tdT^−^ or p21-tdT^+^ HSCs from *p21*-tdTomato mice were transplanted into lethally irradiated (9.5 Gy) B6.SJL (CD45.1) recipient mice in competition with 2 × 10^5^ CD45.1 wBM cells. For noncompetitive BM transplantation, 2 × 10^6^ total BM cells from primary B6.SJL (CD45.1) recipient mice were transplanted into lethally irradiated (9.5 Gy) B6.SJL (CD45.1) secondary recipient mice. For the Zbtb18-knockdown assay, GFP-expressing lentiviruses carrying control or *Zbtb18* shRNA were transduced into C57BL/6J (CD45.2) mouse HSPCs, and 8 × 10^5^ unsorted cells were transplanted into lethally irradiated B6.SJL (CD45.1) recipients or 1.5 × 10^5^ unsorted cells were transplanted into lethally irradiated (9.5 Gy) B6.SJL (CD45.1) recipient mice in competition with 2 × 10^5^ CD45.1 wBM cells.

### Homing assay

10,000 p21-tdT^−^ or p21-tdT^+^ HSCs from *p21*-tdTomato mice (CD45.2) were transplanted into lethally irradiated (9.5 Gy) B6.SJL (CD45.1) recipient mice. Donor-derived CD45.2 cells in the BM of recipient mice were analyzed at 18 h after transplantation.

### Mouse CFU assay

Infected GFP^+^ HSPCs or p21-tdT^−^ and p21-tdT^+^ HSCs were sorted and cultured in methylcellulose-based medium (M3434, StemCell Technologies) in 4–5 replicate wells of 24-well plates. The CFU-G, CFU-GM, CFU-E, and CFU-GEMM colonies were counted and photographed after 7–14 days of incubation.

### RNA extraction and qRT-PCR

Total cellular RNA was isolated with TRIzol reagent (Invitrogen) and used for first-strand cDNA synthesis via the Reverse Transcription System (Roche). Quantitation of all gene transcripts was achieved by qRT-PCR using Power SYBR Green PCR Master Mix (Roche) and Thermo Quant Studio 5 sequence detection system (Thermo), with the expression of *Rpl7* used as the internal control. The primers used in this study were: *Rpl7*, GATTGTGGAGCCATACATTGCA and TGCCGTAGCCTCGCTTGT; *cKit*, GCCACGTCTCAGCCATCTG and GTCGCCAGCTTCAACTATTAACT; *Zbtb18,* CCGCTCCGTGTTATGAAGACA and TGGTCCTTGTAAAAGAGGTGGA; *p21*, ACGGGACCGAAGAGACAAC and CAGATCCACAGCGATATCCA.

### RNA-seq and analysis

p21-tdT^–^ and p21-tdT^+^ HSCs were sorted from the BM of *p21*-tdTomato mice for RNA-sequencing. Total RNA was extracted using TRlzol (Invitrogen). Libraries were prepared using an Illumina RNA library preparation TruSeq PE kit. High-throughput RNA-seq was performed on an Illumina Xten sequencer (paired-end 150-bp sequencing). Clean reads were filtered by removing reads including adapters, reads including poly-N, and low-quality reads from raw data. All the following analyses were based on clean data. Clean data were first aligned to the mouse genome (GRCm38) with GENCODE M16 gene annotation using HISAT2 (Kim et al., 2015). DEG analyses were conducted using Cufflinks and Cuffdiff (v2.2.1) software (Trapnell et al., 2010). Unsupervised hierarchical clustering was conducted using the pheat-map *R* package. Genes with a *q* value < 0.05 in the Cuffdiff results were considered significantly different.

### Cleavage under targets and tagmentation

CUT&Tag experiments were performed as described previously with the Hyperactive *in situ* ChIP Library Prep Kit for Illumina from Vazyme (TD901-01) (Wang et al., 2019). p21-tdT^−^ and p21-tdT^+^ HSCs sorted from BM of *p21*-tdTomato mice and cells were captured with ConA beads and incubated with primary and secondary antibodies in antibody buffer and dig-wash buffer, respectively, for the time indicated in the manufacturer’s instructions. The cells were subjected to incubation with Hyperactive pA-Tn5 Transposon and fragmented within Tagmentation Buffer at 37°C for 1 h. Subsequently, the extracted DNA fragments were subjected to high-throughput sequencing for analysis. Clean reads were aligned to the mouse genome (GRCm38) using the Bowtie2 package (Langmead and Salzberg, 2012), and peaks were detected using MACS2 callpeak (Zhang et al., 2008) with a false discovery rate (FDR) cutoff of 0.05.

### Assay for transposase-accessible chromatin (ATAC) with high-throughput sequencing

ATAC-seq was performed as described previously, In brief, 20,000 p21-tdT^−^ and p21-tdT^+^ HSCs were sorted from the BM of *p21*-tdTomato mice. Cells were lysed in lysis buffer [10 mmol/L Tris-HCl, pH 7.4, 10 mmol/L NaCl, 3 mmol/L MgCl_2_, 0.1% (*v*/*v*) IGPAL CA-630] for 10 min on ice. Then they were centrifuged at 500 ×*g* for 5 min, and the obtained nuclei were added to 50 μL transposition reaction buffer (offered by Vazyme TD501-01) and incubated at 37°C for 30 min. After tagmentation, the reaction was halted using VAHTS DNA Clean Beads, and DNA was purified for final library construction (TruePrep DNA Library Prep Kit V2 for Illumina) before paired-end high-throughput sequencing using HiSeq XTe. Clean reads were aligned to the mouse genome (GRCm38) using BWA package (Zhang et al., 2008) and peaks were called using MACS2 package with an FDR cutoff of 0.05.

### Luciferase reporter assay

The modulation of *cKit* gene promoter repression by p21 and ZBTB18 was analyzed by luciferase assay using 293T cells. The region from −1,500 to +500 bp distance upstream of the transcription start site of the *cKit* gene was synthesized and cloned into a pGL3-basic-luciferased vector. Luciferase reporter activity was measured using the Dual Luciferase Assay System (E1910; ProMega). Relative luciferase activity was normalized to Renilla luciferase and control vector luciferase reporter activity.

### Statistical analyses

At least three independent replicates were included for all functional experiments. FCS (flow cytometry standard) files were analyzed using Flow Jo software (Flow Jo LLC,650 Ashland, OR, USA). GraphPad Prism v8.4.0 software was used for statistical analysis and generating graphs. Data are presented as the means ± the standard deviation (SD). An unpaired two-tailed Student’s *t* test was used to compare two groups of data. Survival was analyzed by a Log-rank test. Two-way ANOVA with Geisser-Greenhouse correction was used to compare multiple groups of data. A *P* value < 0.05 was considered statistically significant.

## Supplementary data

The online version contains supplementary material available at https://doi.org/10.1093/procel/pwae022.

pwae022_suppl_Supplementary_Figures_S1-S4

pwae022_suppl_Supplementary_Table_S1

pwae022_suppl_Supplementary_Table_S2

pwae022_suppl_Supplementary_Table_S3

pwae022_suppl_Supplementary_Table_S4

pwae022_suppl_Supplementary_Table_S5

pwae022_suppl_Supplementary_Table_S6

## Data Availability

RNA, cleavage under targets and tagmentation (CUT&Tag), and transposase-accessible chromatin (ATAC) sequencing data have been deposited in the NCBI SRA (PRJNA1021347, PRJNA1018269).

## Code availability

This paper does not report original code.
