# Supplementary material for: p21/Zbtb18 repress the expression of cKit to regulate the self-renewal of hematopoietic stem cells
Source: Protein Cell. 2024 May 9;15(11):840–57. doi: 10.1093/procel/pwae022 (PMC11528518; doi:10.1093/procel/pwae022)
Supplement: pwae022_suppl_Supplementary_Figures_S1-S4 [file pwae022_suppl_supplementary_figures_s1-s4.pdf]

Supplementary Figure Legends:

Supplemental Figure 1. p21-tdT^{+} HSCs were in an inactive state of cell division

(A) Generation of *p21*-tdTomato knock-in allele. (B) FACS gating strategies for indicated cells. (C) Representative flow cytometric analysis showing the percentages of p21-tdT^{+} cell populations in Lin^{+}, Lin^{-}, LKS^{-}, CMP, GMP, MEP, and MkP from wild type (WT) and *p21*-tdTomato mice. (D-E) Representative flow cytometric analysis (D) and the histograms (E) showing the percentages of p21-tdT^{+} cell populations in CD3^{+}, B220^{+}, CD11b^{+} and Ter119^{+} cells from wild type and *p21*-tdTomato mice. (F) The kinetics of cell division in p21-tdT^{-} and p21-tdT^{+} HSCs during the 4-day culture period.

Supplemental Figure 2. p21-tdT^{+} HSCs possess enhanced self-renewal capacity

(A) Representative images of single-cell culture clones. Scale bars, 5mm. (B) Representative images of typical clones from single-cell culture. Scale bars, 500μm. (C) Representative images of cell population cultures. Scale bars, 200μm. (D) Percentages of different colony types in primary culture on day 8. (E-F) Representative flow cytometric analysis (E) and the histograms (F) showing the percentages of donor-derived CD45.2 cells in the BM of recipient mice at 18h after transplantation (n = 5 recipient mice per condition). (G) Percentages of donor-derived LT-HSCs in the BM of recipient mice in 4th month after primary transplantation (n = 4 recipient mice per condition). (H) Representative flow cytometric analysis showing the gating strategies for the indicated donor-derived cells. Data are represented as mean ± SD. ns, No Significance; *, $P < 0.05$; **, $P < 0.01$; unpaired two-tailed Student's t test for D.

Supplemental Figure 3. Zbtb18 is highly expressed in p21-tdT^{+} HSCs

(A) Heatmap showing ATAC-seq results for p21-tdT^{-} and p21-tdT^{+} HSCs from *p21*-tdTomato mice. (B) TSS profile and heatmap of CUT&Tag sequencing results showing binding of H3K4me1, H3K4me2, H3K4me3 histone modifications in p21-tdT^{-} and p21-tdT^{+} HSCs.

Supplemental Figure 4. p21 interacts with ZBTB18 to repress the transcription of *cKit*

(A) Representative gating strategy for flow cytometric analysis of cKit expression in p21-tdT^{-} and p21-tdT^{+} HSCs (LKS^{+}CD150^{+}CD48^{-}) from *p21*-tdTomato mice. (B) Representative gating strategy for flow cytometric analysis of p21 expression in cKit-high and cKit-low HSCs (LKS^{+}CD150^{+}CD48^{-}) from *p21*-tdTomato mice. (C) Western blotting analysis showing the expression of indicated proteins in the reporter assays of 293T cells. (D) The summary about the mechanism and function of p21 in HSCs is as follows: p21 can interact with ZBTB18, co-repressing the expression of cKit with decreased chromatin accessibility. Therefore, p21-tdT^{+} HSCs, characterized by low expression of cKit, demonstrate enhanced long-term and self-renewal abilities compared to p21-tdT^{-} HSCs.

Table S1. Differentially expressed genes in p21-tdT^{-} and p21-tdT^{+} HSCs

Table S2. ATAC-seq upregulated genes in p21-tdT^{+} HSCs (76)

Table S3. Differentially expressed genes of H3K27ac CUT&Tag sequencing in p21-tdT^{-} and p21-tdT^{+} HSCs

Table S4. Zbtb18 CUT&Tag sequecing upregulated genes in p21-tdT^{+} HSCs (8968)

Table S5. H3K4me3 CUT&Tag downregulated genes in p21-tdT^{+} HSCs (663)

Table S6. Overlapped genes of upregulated genes in Zbtb18 CUT&Tag-seq and downregulated genes in RNA-seq, H3K4me3 and H3K27ac CUT&Tag-seq in p21-tdT^{+} HSCs (47)

sFigure 1

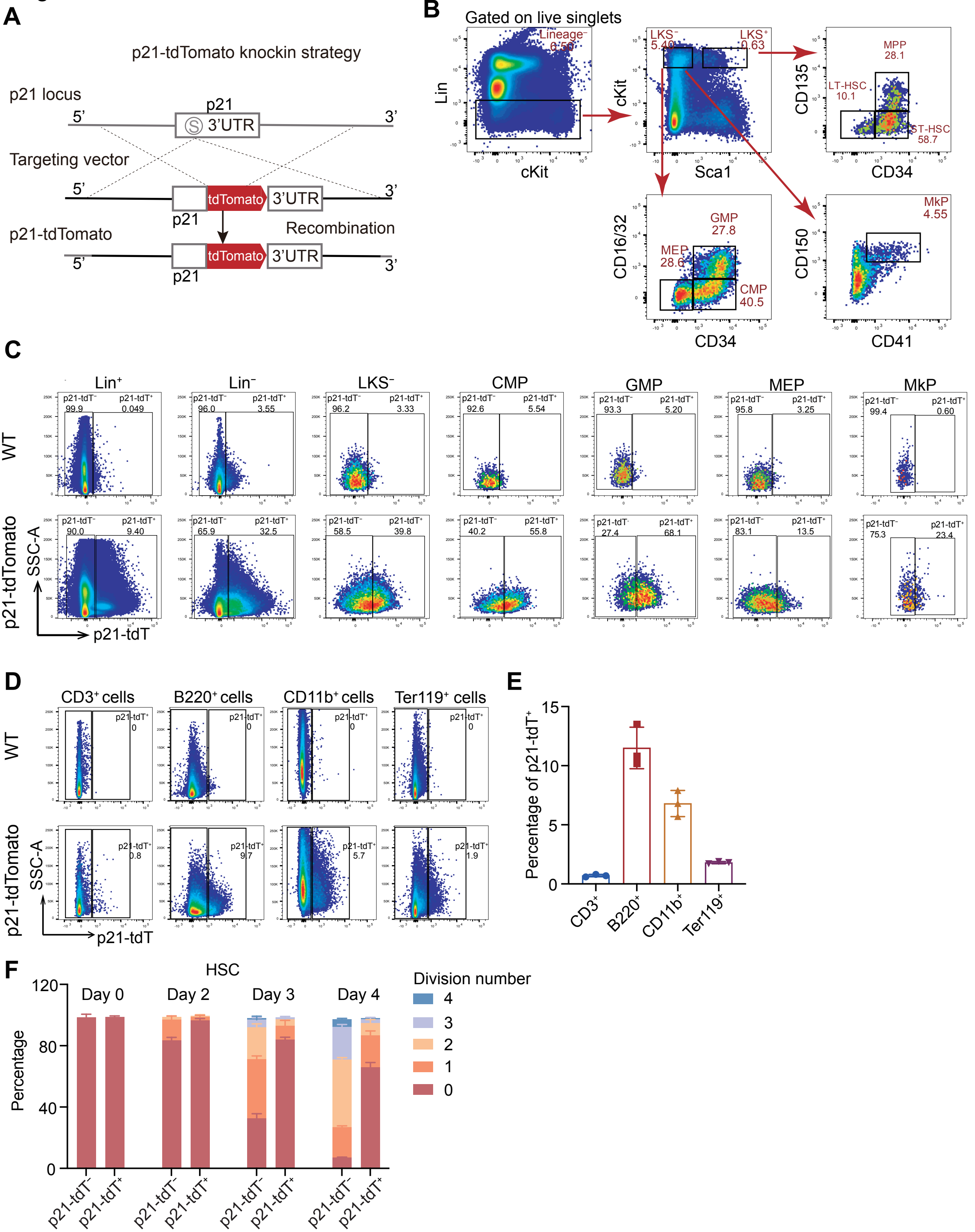
A
p21-tdTomato knockin strategy
p21 locus
p21
3'UTR
5'
3'
Targeting vector
tdTomato
p21
Recombination
p21-tdTomato
B
Gated on live singlets
Lineage⁻
6.50
Lin
cKit
LKS⁻
5.40
LKS⁺
0.63
Sca1
MPP
28.1
LT-HSC
10.1
ST-HSC
58.7
CD135
CD34
GMP
27.8
MEP
28.6
CMP
40.5
CD16/32
MkP
4.55
CD150
CD41
C
Lin⁺
Lin⁻
LKS⁻
CMP
GMP
MEP
MkP
WT
p21-tdTomato
SSC-A
p21-tdT
D
CD3⁺ cells
B220⁺ cells
CD11b⁺ cells
Ter119⁺ cells
E
Percentage of p21-tdT⁺
F
HSC
Day 0
Day 2
Day 3
Day 4
Percentage
Division number
p21-tdT⁻
p21-tdT⁺

sFigure 2

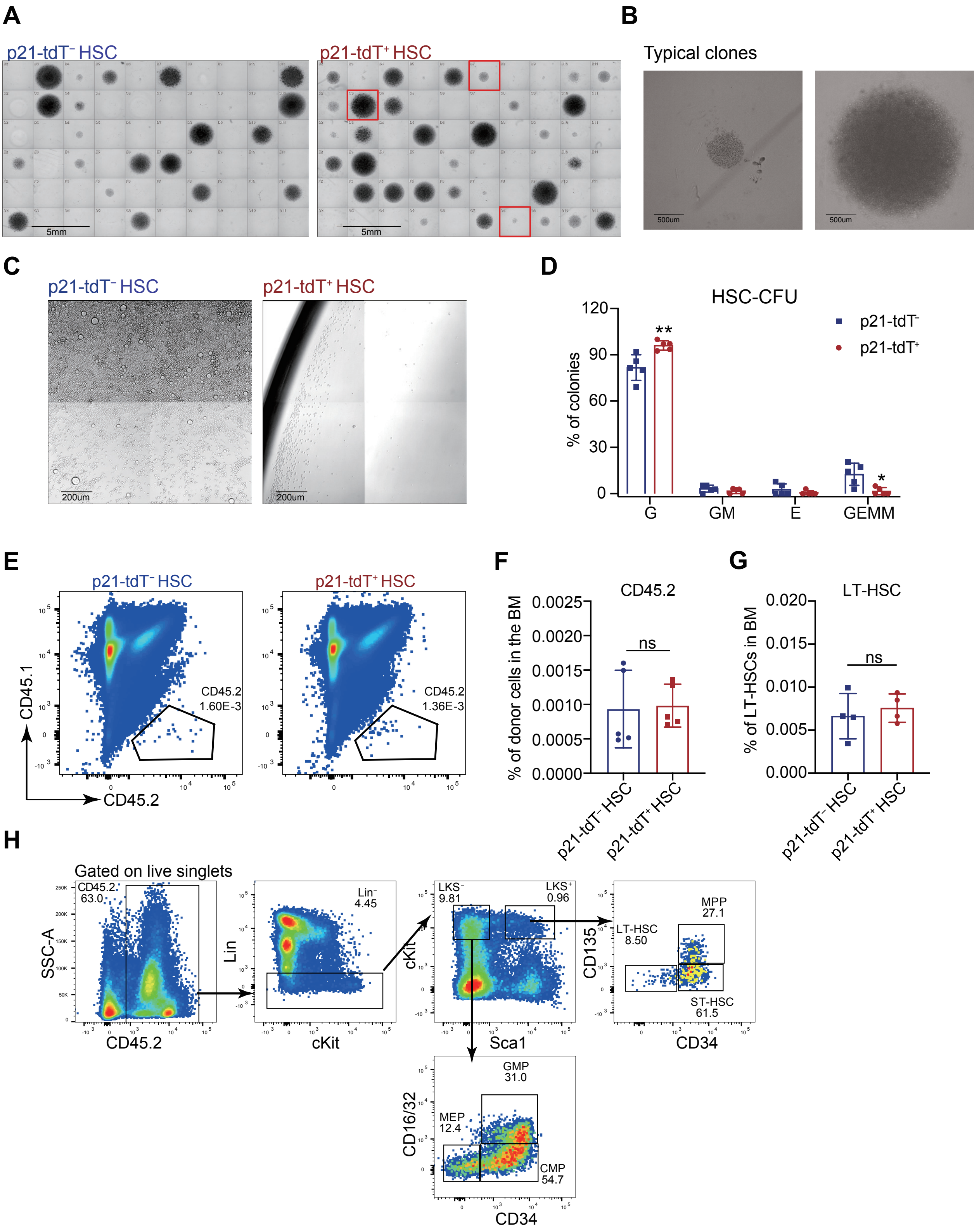

A
p21-tdT⁻ HSC
p21-tdT⁺ HSC
5mm
5mm
B
Typical clones
500um
500um
C
p21-tdT⁻ HSC
p21-tdT⁺ HSC
200um
200um
D
HSC-CFU
% of colonies
p21-tdT⁻
p21-tdT⁺
G
GM
E
GEMM
E
p21-tdT⁻ HSC
p21-tdT⁺ HSC
CD45.1
CD45.2
CD45.2 1.60E-3
CD45.2 1.36E-3
F
CD45.2
% of donor cells in the BM
ns
p21-tdT⁻ HSC
p21-tdT⁺ HSC
G
LT-HSC
% of LT-HSCs in BM
ns
p21-tdT⁻ HSC
p21-tdT⁺ HSC
H
Gated on live singlets
SSC-A
CD45.2 63.0
CD45.2
Lin
Lin⁻ 4.45
cKit
LKS⁻ 9.81
LKS⁺ 0.96
Sca1
CD135
LT-HSC 8.50
MPP 27.1
ST-HSC 61.5
CD34
CD16/32
GMP 31.0
MEP 12.4
CMP 54.7
CD34

sFigure 3

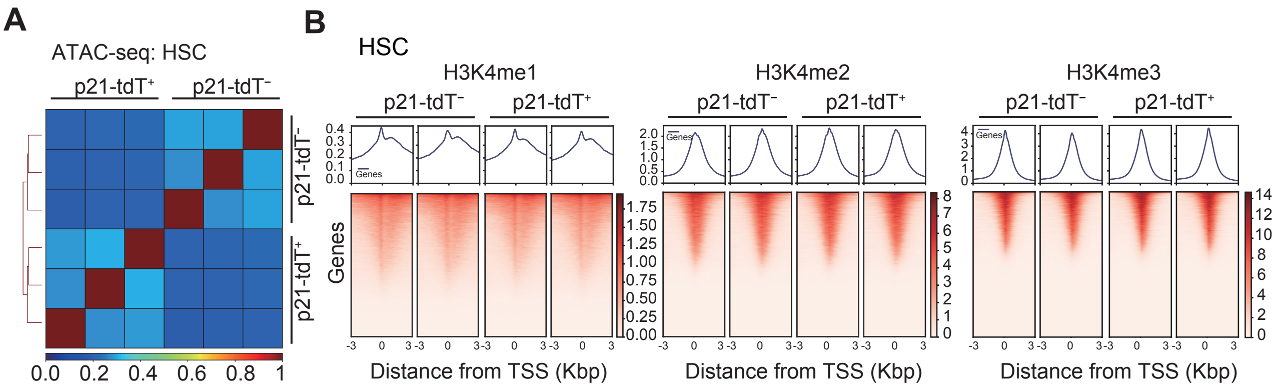

A
ATAC-seq: HSC
p21-tdT+
p21-tdT−
p21-tdT−
p21-tdT+
0.0 0.2 0.4 0.6 0.8 1
B
HSC
H3K4me1
H3K4me2
H3K4me3
p21-tdT−
p21-tdT+
Genes
Distance from TSS (Kbp)

sFigure 4

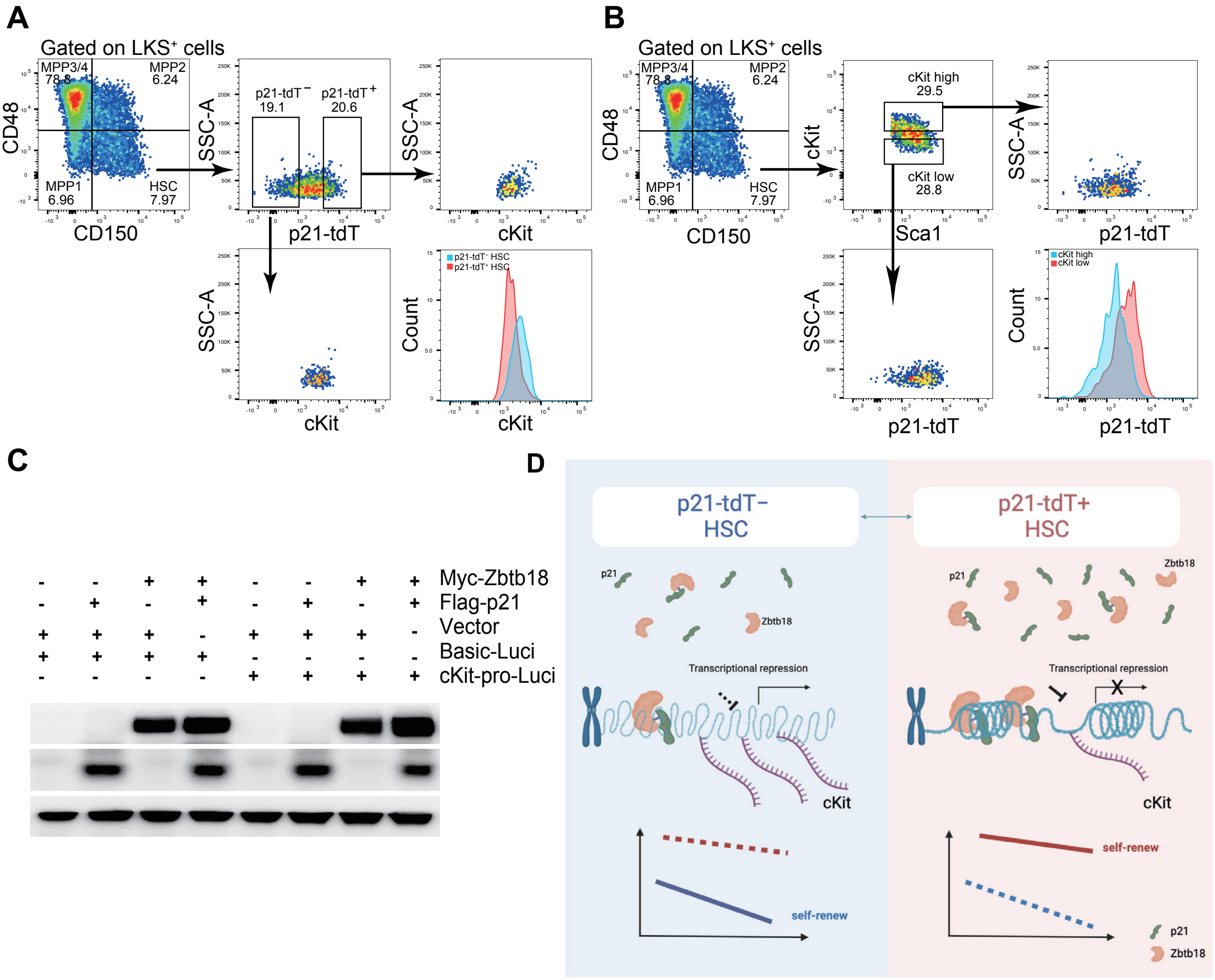
A
Gated on LKS⁺ cells
MPP3/4
78.8
MPP2
6.24
MPP1
6.96
HSC
7.97
CD48
CD150
SSC-A
p21-tdT⁻
19.1
p21-tdT⁺
20.6
p21-tdT
cKit
Count
p21-tdT⁻ HSC
p21-tdT⁺ HSC
B
Gated on LKS⁺ cells
cKit high
29.5
cKit low
28.8
Sca1
cKit high
cKit low
C
Myc-Zbtb18
Flag-p21
Vector
Basic-Luci
cKit-pro-Luci
D
p21-tdT–
HSC
p21-tdT+
HSC
p21
Zbtb18
Transcriptional repression
cKit
self-renew
